# On Dr. Brown-Sequard and His Recent Lectures

**Published:** 1878-06

**Authors:** J. S. Jewell

**Affiliations:** Professor of Nervous and Mental Diseases in the Chicago Medical College


### Chicago Medical Journal

AND

### EXAMINER.

VOL. XXXVI.—JUNE, 1878.—No. 6.

### Original Lectures.

### ON DR. BROWN-SÉQUARD AND HIS RECENT LECTURES.

A LECTURE DELIVERED BEFORE THE CHICAGO MEDICAL SOCIETY, MAY 6TH, 1878.

BY J. S. JEWELL, M. D.,

(*Professor of Nervous and Mental Diseases in the Chicago Medical College.*)

MR. PRESIDENT AND MEMBERS OF THE SOCIETY:

This evening it is my purpose to review briefly certain of the positions taken by Dr. Brown-Séquard, in relation to the physiology and pathology of the nervous system, more particularly as set forth in a course of lectures delivered by him recently in this city. I do this for several reasons: because I deem many of the positions taken thoroughly erroneous, because they seem to be in direct variance with the best teachings upon the anatomy and physiology of the nervous system, because these erroneous notions are stamped with the great authority of Dr. Brown-Séquard, and because they were delivered before an unusually large audience of medical men, such as but few living persons could call together for the discussion of such a subject, and hence in so far as the revolutionary views referred to may have been adopted by his audience, it may be assumed that the progress of a true knowledge upon the important theme to which his lectures related, may have been retarded.

No one has a greater measure of well-founded respect for Dr. Brown-Séquard than I have. For years I have read his writings and tried to understand his various and valuable contributions to physiology and physiological pathology. To untiring industry he has united great ingenuity in devising his experiments, far more than an ordinary caution in adopting conclusions, and no ordinary knowledge of the sources of error and conditions of success in physiological experimentation. In his earlier writings he is clear and consecutive in thought and statement, simple in style, and convincing in argumentation. In spite of a restless and unsettled disposition which has led him almost perennially from the United States to France, and from France to England, or *vice versa,* in spite of a comparative lack of pecuniary resources, and finally in spite of the fact that he has not been, by reason of frequent removal, long at a time able to occupy a professorial chair, which would have yielded a support and the means and leisure to prosecute scientific research, I say that in spite of these drawbacks, he has left indelibly his impress on the annals of physiology and medicine.

It is not my purpose this evening to try to recount the various contributions to physiology made by Dr. Brown-Séquard, even in relation to the nervous system. This will be a work for the future. His researches on the physiology of the spinal cord, constitute his chief and sufficient title to a place in the front rank of physiologists. The researches of no other physiologist in regard to the modes of action of the nervous system, no matter from what standpoint we may regard them, are entitled to a higher place. Then his researches into the physiology of the vaso-motor nervous system, into the nature of epilepsy, not to mention the trophic and temperature relations of the nervous system, are among his more prominent contributions to physiological science. By them he has been greatly honored, and will be always remembered. But it must be remembered that physiology is even now a very imperfect science. Much as is now known, much is yet to be learned in the future.

Until within the past few years, Dr. Brown-Séquard was in accord with the general current of physiological thought. But for seven or eight years or about that time, he has abandoned many of his former notions, and is now apparently in almost complete disaccord with the latest, and what seem to be the best results of anatomical and physiological science, more particularly as regards the brain. The first general indication I remember to have seen of his change of view, was in the *Archives of Scientific and Practical Medicine*, a monthly journal which was started in New York several years ago, with Dr. Brown-Séquard as its editor, and which barely reached an existence of half a year and then expired. In that journal there was an article or two in which, in the form of a series of propositions, certain rather startling innovations in nerve physiology were promised. But the sudden decease of the journal, arrested their publication at that time. A little later, a lecture by Dr. Brown-Séquard was published in the *Boston Medical and Surgical Journal* for July 19, 1875, in which his promise was partly fulfilled. In that lecture, he especially devoted himself to the task of combatting the doctrine of a localization of function in the cortex of the brain, which subject had then begun to attract attention, through the earlier researches of Fritsch and Hitzig, and Dr. Ferrier. Soon afterwards he was found in Paris, where he became engaged almost by accident in a very elaborate discussion in the Biological society of that city, with Dr. Charcot, Dr. Luys, and others, all or most of whom took ground against him, Dr. Charcot from the clinical side, Dr. Luys from the anatomical, others from the physiological, and all with a result anything but favorable to Dr. Brown-Séquard. These discussions were reported at some length and discussed in my journal.

Subsequently Dr. Brown-Séquard went to London, and delivered lectures on the same subject, which were published in the *London Lancet*. Not long after this, lectures in substance the same, were delivered at New York and reported for the *Medical Record* of that city.

In all these lectures and discussions the chief themes were a denial of the localization of functions in the cerebral cortex, an assertion of the doctrine rather than the fact, that motor conductors do not decussate at or below the "base of the brain," and finally the unlimited use of the principle of "inhibition," and its contrary, as a means for enabling him to explain a host of knotty questions in the physiological pathology of the brain. This has been the burden of all his recent lectures on these subjects, especially of those he gave in this city. To those who have been closely watching his course the past few years, his peculiar views as regards the physiology of the brain have ceased to attract serious notice. They are not shared, as a whole, by a single physiologist of eminence. But few clinicists and not one of marked prominence, fully shares his opinions. The anatomists are almost all against him or at least not for him. It must be remembered that Dr. Brown-Séquard is not an anatomist in the sense that he is a physiologist. In the latter province he is an authority, in the former he is not so. Since then, his peculiar views which have been so often published, and judged by the present notions which prevail in the physiological anatomy of the nervous system as I hope to show, are so far from the truth, it was with some surprise that I learned the *Chicago Medical Journal and Examiner*, usually so enterprising and alert, had concluded to publish the lectures.

The first question to which I would invite your attention is that of the decussation of the conductors of motor impressions, extending from the base of the brain downwards into the spinal cord. The common opinion has been, and now is, that motor fibers start—let us suppose—in the striate body in one side of the brain, and these pass downwards in the crus of the cerebrum into the pons varolii of the same side, and then either in the pons or the medulla, or both, they (the bands of motor conductors) cross over to the opposite side, so as to enter the opposite side of the cord, in such manner that the fibers—or at any rate, the same motor conductors—which arise in the right half of the brain, are found lower down, in the left half of the spinal cord, and so for the left half of the brain and the right half of the spinal cord. This I say has been and now is the common opinion, but it does not seem to be the opinion of Dr. Brown-Séquard. I will make a few extracts from his lectures, which go to show what are his real views on this subject. Says he (*Journal and Examiner, p.* 292, *March,* 1878): "Since the time that paralysis has been observed to come usually in the side of the body which is opposite to that of the seat of the lesion in the brain, it has been considered that one side of the brain is the mover of the opposite of the body. *This I will contradict absolutely.*" "It was admitted for a long time ago * * * that if paralysis comes on the opposite side of the disease in the base of the brain, it exists on the opposite side because decussation takes place only in the anterior pyramid, and produces paralysis on the opposite side, though there are exceptions. I will try to prove * * that *the views I have to propose are absolutely different from these.*" (P. 294.) The meaning of these passages and many more of similar import seems to be, that there is no decussation of motor conductors below "the base of the brain," as is almost universally held at this hour among competent anatomists, physiologists and clinicists. Let us examine this question with some little care, to see in what state it is.

That as a rule and as a whole, the conducting motor tracts from one side of the brain, decussate below the brain, so as to enter the opposite half of the cord, is rendered highly probable by the following:

1. *Anatomical Evidence.* Of course it will be impossible for me to enter now into this or any other kind of evidence in much if any detail, for such a course would require a course of lectures, rather than one lecture. But not to refer to any other writings or researches at present, I would call to your minds the comparatively recent work done by MM. Sappey and Duval, in regard to the fine anatomy of the medulla and pons, and "the course of the columns of the cord" through the same, as reported in various journals for the year 1876 (*Gaz. des Hôpitaux,* No. 10, Jan. 25; also, *Journal of Nervous and Mental Disease,* p. 324, vol. iii., 1876). No anatomical researches on these parts were ever conducted with more care or skill, or by more capable observers, and the results are clear and absolute as to the decussation of the conducting motor tracts in the medulla, but *only in part* where Dr. Brown-Séquard seems to assume that they pass, viz.: in the superficial or exposed parts of the anterior pyramids. Many if not most of the fibers (or routes, whether fibers or cells) seem to decussate more deeply in the medulla. Hence the lack of value of many of the cases of superficial disease of the medulla and pons, on which Dr. Brown-Séquard seems to rely to prove that there is not a decussation of motor conductors, where it had been formerly supposed solely to occur. But more of the applications of anatomical facts hereafter.

Then again the same subject has been carefully examined from many points of view, by Dr. Flechsig, of Leipsic in a recent work (*Die Leitungsbahnen im Gehirn und Rueckenmark des Menschen auf Grund Entwicklungsgeschichtlicher untersuchungen dargestellt von Dr. P. Flechsig. Mit* 20 *Tafeln.* p. 383 Leipzig, 1876,) and in subsequent publications his views are essentially confirmed. The conducting motor tracts according to Dr. Flechsig *do* decussate, with certain partial and rather rare exceptions (in man). In fact the *anatomical* evidence worthy of the name is all in favor of the decussation. I could fill page after page, with quotations from the works named, and many others not less respectable and conclusive, but I do not have time to go into details. This statement is not made, of course, as an excuse for not giving the facts *in extenso*, for, they can be produced when time and opportunity admit. The anatomical evidence is conclusive, as far as could be expected.

2. *Physiological evidence.*—This is quite extensive and at the same time, in the main, conclusive. I can do no more now than refer to that which seems most important. First of all I would refer to certain facts which it may be said Dr. Brown-Séquard himself, discovered. I now refer to his researches on the spinal cord. Among other facts established by his researches are the following:

If a complete lateral hemi-section of the cord is made, in any part of its course, let us suppose at the summit of the cord prior to its transition into medulla, the following circumstances among others, invariably appear; paralysis of the motion of the muscles of the members on the same side as that on which the cut is made, or at least paralysis of all muscles on the same side, which receive their motor nerves from the cord below or behind the section, and a corresponding paralysis of sensibility on the *other side* of the body—or that opposite to the cut, and to the motor paralysis. These phenomena invariably occur as consequences of a complete lateral hemi-section. They were very properly held by Dr. Brown-Séquard, to indicate that the sensory fibers of the spinal nerves (posterior roots) decussate in the cord, soon after they enter it, so that the sensory nerve fibers which go from the right side of the body, cross over the middle line and ascend in the left half of the spinal cord towards the brain. This fact was established in various ways, and is now no longer in doubt. The sensory conducting tracts, *do decussate* at all heights in the cord, soon after they have entered it, from the posterior roots of the spinal nerves. Do the motor conducting tracts also decussate in the cord, following the example of the sensory tracts? Most certainly not. The same experiments which prove that the sensory tracts do discussate, prove in the most conclusive manner that motor conductors do not. If they decussate at all it must be above the cord, for in the latter, the sensory conductors for one side of the body and the motor conductors for the other, are found on the same side of the cord. Three courses of supposition are now open to us: Either the sense conductors from the right side of the body go to one side of the brain, and the motor conductors for the right side of the body go from the other side of brain, as compared with the sense conductors, or the sense conductors after having crossed over in the cord, cross back again "at the base of the brain," so that the sense and motor conductors for one side of the body may be at last conjoined in the brain as they are separated or on opposite sides in the cord, or finally we must suppose, that there is a decussation of motor conducting tracts above the cord and below the brain. We must adopt one or other of these views. The first no one would think of maintaining, save in the most exceptional cases, in the present state of nerve physiology. The second supposition would be even more difficult than the former to reconcile to our existing knowledge. The third supposition is the common one entertained to day, and has been for a long time past. Besides these suppositions, I do not know of any others which are even probable. This latter supposition is strongly supported, by many well established facts both in physiology and pathology.

If Dr. Brown-Séquard has collected "right and left," two hundred cases of brain disease causing paralysis on the same side as the brain lesion, three times that number, at any moderate estimate can be shown, in which the opposite is true—lesion in one side of the brain with paralysis in the opposite half of the body, and among these cases are very many, in which the same lesion in one side of the brain has caused not only motor paralysis on the opposite side of the body, *but also a sensory on the same side as the motor paralysis.* This class of facts is well known, and shows that though the sensory and motor conductors for one side of the body, are on opposite sides of the cord, *that when we come to the brain they are on the same side, and hence there must have been a decussation above the cord.*

Then again it is true, that in experiments on the brains of living monkeys, not to mention animals below them, experimental injuries made on the right side of the brain for example have in almost every case been followed by paralysis or convulsions, *in the opposite side of the body.* This has been the uniform experience of almost every one, save Dr. Brown-Séquard. Dr. Ferrier, of London, who has conducted a greater number of experiments on monkeys than any other man, which animals so nearly approach man in their nervous organization, *has always found* paralysis to occur on the side opposite to the lesion. What do such experiments oblige us to suppose? A decussation of motor fibers, (or conducting tracts) to the opposite side, which is what Dr. Brown-Séquard strangely enough seems to deny.

Here again pathological cases are exceedingly numerous, twenty to one of Dr. Brown-Séquard's cases, which show in all desirable plainness that a lesion in the base of one side of the brain causes motor disturbances in the opposite half of the body. And many of these cases are among the most recent and most carefully observed. They so far outweigh in number and trustworthiness the cases relied on by Dr. Brown-Séquard, in support of his views, that one is led very naturally to consider his cases as either inaccurately reported, or simply anomalous. Now what are the facts on which Dr. Brown-Séquard relies, to induce us to set aside the evidence I have hinted at, as supporting the view that a decussation of motor conductors takes place above the cord, and below the brain?

In the *London Lancet*, (*On the Appearance of Paralysis on the side of a Lesion in the Brain, by C. E. Brown-Séquard*) for April, 1876, is a lecture, by Dr. Brown-Séquard, in which he gives the literary references to many of his cases. In that lecture he says, "I may startle many of my hearers in stating that I have collected *more than two hundred* of such cases [of paralysis on the same side as the lesion.] Burdach, out of 258 cases of paralysis in one-half of the body, states that there were fifteen on the side, and 243 on the opposite side of a lesion in the brain. W. Nasse, besides the fifteen cases of Burdach, knew of 26 cases of paralysis on the injured side in the brain. In two good papers by Bayle and Dechambre, not more than ten or twelve cases are pointed out. The 200 I have collected do not include those of Nasse's list (at least most of them), as I have not been able to procure the paper of that learned physician." (P. 146 *Lancet.*)

But the 200 cases of Dr. Brown-Séquard in all probability include those of Burdach, Bayle, Dechambre, Gintrac, etc. Now I have been at the pains to examine the reports of many of these cases, and among them those of the paper of Nasse (*Ueber die sogenannte gleichseitige Hemiplegie. Von Werner Nasse, in Bonn. Allgem. Zeitschr. f. Psych. etc.* Bd. 6, 1849, S. 384–412), especially to ascertain the sources from whence they drew their materials. The first cases are from the work of Morgagni (*De Sedibus et Causis Morborum, Op. lvii.*, 14). Valsalva and Morgagni, it seems, were able to collect eight original cases in all. Next Bayle, in 1824, published a paper, in which seven cases were mentioned, *six of them the same* as had been already mentioned by Morgagni. Then, of the fifteen mentioned by Burdach, two of them, at least, occur in Morgagni's list, while of the ten cases cited by Dechambre, *eight had been already mentioned by either Morgagni or Bayle.* Of the cases published by Andral —sixteen in number—*nine had been already mentioned* by one or other of the earlier writers mentioned, leaving only seven new cases, and two of them oral.

Thus we have 32 cases, and to this number 26 are added by Nasse, after a most painstaking review of medical literature.

Nasse's collection added to the previous one gives us 58, so that either Dr. Brown-Séquard must have found some new cases prior to 1849, or since then he must have found in more recent literature about 150 new cases. But from the references contained in the lecture in the *Lancet*, it would appear that most of the cases among the 200 are old, and as I know from having read the original accounts of many of them, are very imperfectly reported, and are hence absolutely devoid of solid scientific value. I have no fear whatever that this statement will be successfully contradicted by a recital of the cases. As it is obviously impossible to go far into details, I will limit myself to two of the best and most recent cases, depended on to show there is not a decussation of the motor conductors in the medulla. Assuming that the place of decussation of motor conductors is between the anterior pyramids of the medulla, and there alone, Dr. Brown-Séquard quotes two cases from Prof. Vulpian (see his *Leçons sur la Physiologie Générale et Comparée du Système Nerveux, etc.*, *Paris*, 1866. P. 492–494), of disease of the anterior pyramids, without the paralysis being present which should have been, if the anterior pyramids had contained the motor conductors, as was formerly supposed.

I will recite the best one of these cases *verbatim*, so you can see for yourselves what it amounts to. The first was as follows: "In a woman aged 82 years, and who showed no appreciable paralysis, the autopsy (no account of what the woman actually died of) revealed the evidence of a lesion of the pons and of the anterior pyramids. There was at the anterior part of the under surface of the pons an irregular, hemispherical cavity, with grayish walls, about half a centimetre in diameter, and from five to six millimetres in depth, and containing a transparent liquid. Beyond this, there was *a very slight* loss of substance, on the inferior surface of the left cerebral peduncle, five or six millimetres from the anterior edge of the protuberance. The two anterior pyramids presented a manifest atrophy, more deep on the right than the left. On the left side the lesion was very superficial, involving only the external part of the pyramid, where there was to be seen a very small, narrow band, of a grey color, formed by altered fibers. But the atrophy of the right pyramid *appeared* to be complete, its prominence being much diminished and being of a grayish yellow color in its whole thickness. *No microscopic examination was made* of such a character as to determine whether or not there were yet healthy fibers."

The other case is not so important even as this one, except that a more careful microscopic examination was made. But take the case I have just quoted and consider it a moment. *It is not certain* that the whole thickness of the right pyramid was destroyed, and the left is known not to have been. But if it had been, the whole of the motor conducting tract, according to our present knowledge, would not have been destroyed, and hence the case, though interesting, is, for exact scientific purposes, worthless. And I speak advisedly when I say to you that there is probably not one of his 200 cases free from serious objections of a similar kind. What kind of a basis, then, does this afford for founding a new and revolutionary doctrine, such as Dr. Brown-Séquard has announced? The cases collected by Burdach were 258 in number (*Vom Baue und Leben das Gehirn Bd. iii.* S. 368. Leipzig, 1826), and *only fifteen* appeared to be cases where paralysis was on the same side as that of the lesion—*or one case in seventeen of hemiplegia.* So, according to the old table of Burdach, the cases on record of crossed as compared with direct hemiplegia, were as 17 to 1. I am prepared to say now, that there are 30 cases to one at present on the records. If so what shall we think when it is learned that none of Dr. Brown-Séquard's cases are critical when many of the other kind are critical. The immense preponderance of the crossed to the direct cases, is alone sufficient to throw a fatal suspicion on the one case which appears to be different from the other thirty.

[TO BE CONTINUED.]

---

A NEW MUCILAGE.—The *Journal de Pharmacie* states that if, to a solution of gumarabic, measuring $8\frac{1}{3}$ fluid ounces, a solution of 30 grains of sulphate of aluminum, dissolved in two-thirds of an ounce of water, be added, a very strong mucilage is formed, capable of fastening wood together, or of mending porcelain or glass.

